# The roles of churches in HIV prevention among youth at Nqutu in KwaZulu-Natal South Africa

**DOI:** 10.4102/curationis.v46i1.2442

**Published:** 2023-05-15

**Authors:** Siphiwe T. Madlala, Sethembile Khanyile

**Affiliations:** 1 Department of Nursing Science, Faculty of Science, Agriculture and Engineering, University of Zululand, Empangeni, South Africa

**Keywords:** churches, church youth, church leaders, church organisation, HIV and/or AIDS

## Abstract

**Background:**

Human immunodeficiency virus (HIV) and acquired immunodeficiency syndrome (AIDS) is a crisis of massive economic, social, spiritual, as well as political magnitudes. Joint United Nations Programme on HIV/AIDS (UNAIDS) revealed that in 2018, there were 37.9 million individuals across the globe who lived with HIV and/or AIDS. The religion is an essential tool used by the pastors to help people living with HIV to come to terms with the illness. Therefore, it is imperative that religious leaders should be actively involved in the prevention of HIV epidemic among the youth.

**Objectives:**

The study objective was to explore and describe the roles of churches in HIV prevention among youth at Nqutu in KwaZulu-Natal.

**Method:**

A qualitative descriptive phenomenology design was employed. Data were collected from 18 to 35 years old participants who were purposefully sampled. Unstructured interviews were conducted to collect data, which was determined by saturation and analysed using Colaizzi’s method of data analysis.

**Results:**

The study revealed five major themes, which consisted of churches’ contribution to HIV prevention; health awareness; churches’ involvement in sexual education; churches’ partnership with stakeholders, human and financial resources. It became evident that churches’ contribution to HIV and/or AIDS prevention is beneficial to the youth.

**Conclusion:**

The study’s findings revealed that churches play a significant role in HIV prevention among youths. Programmes available in churches play an important role among the youth in curbing the HIV epidemic.

**Contribution:**

The Department of Health should consider collaborating with church leaders to promote and prevent HIV and/or AIDS among youth.

## Introduction

The human immunodeficiency virus (HIV) and acquired immunodeficiency syndrome (AIDS) remain the most devastating disease humankind has ever faced. It is a menace to vital human development as well as to the mission of religious organisations. Based on the reports by Joint United Nations Programme on HIV/AIDS (UNAIDS), in 2018, there were 37.9 million individuals across the globe who were living with HIV (World Health Organization [WHO] 2018). The report further indicates that 23.3 million individuals accessed antiretroviral therapy, while another 1.7 million individuals were newly infected with the virus (WHO 2018). At the same time, about 770 000 individuals died as a result of AIDS-related diseases (UNAIDS 2018). The report also observed that about 74.9 million individuals are infected with the virus since its advent in 1981 approximately 32.0 million individuals have died because of AIDS-related diseases since the beginning of the epidemic (UNAIDS 2018). Almost a fourth of South African women in their reproductive ages (15 - 39 years) are HIV-positive. The total number of persons living with HIV in South Africa increased from an estimated 3.8 million in 2002 to 8.2 million by 2021 (Kanda et al. 2013). There is a need for religious leaders to involve their churches and communities to tackle the HIV epidemic (Schoeman 2017). As a result of their prestige in society, churches could have a substantial influence on HIV and/or AIDS prevention. The UNAIDS (2018) corroborated that churches are included as important contributors in the fight against HIV. Churches have strengths and credibility from the community and the members of the church (Ochillo, Van Teijlingen & Hind 2017).

The early collaboration of religious organisations in combating HIV and/or AIDS in Africa has been identified as a crucial and replicable influence in HIV and/or AIDS reduction elsewhere (Errikson, Lindmark & Haddad 2014). The numbers of HIV-positive cases among the youth are increasing daily. In the sub-Saharan Africa, about four in five new infections among adolescents who are aged 15–19 years occur among girls, and furthermore, young women who are aged 15–24 years are twice as likely to live with HIV in comparison to men (UNAIDS 2018). Despite advances in HIV research in terms of treatment and prevention, many people who are living with HIV or at risk of contracting HIV still lack access to prevention, care and treatments (UNAIDS 2018). On the same note, there is still no cure for the HIV epidemic. It should, however be observed that effective treatment using antiretroviral drugs can control the virus so that individuals who are on treatment continue to live longer and healthy lives.

Reducing sexual transmission of HIV is a major challenge. Despite wide scale improvement in HIV prevention efforts through male and female condom promotion, treatment of sexually transmitted disease, HIV counselling and testing, risk reduction counselling and medical male circumcision, HIV statistics among youth remains high (Karim et al. 2012). The churches are grounded in the communities and as a result they can play a significant role in the fight against HIV. In the nations with a high prevalence of HIV, churches could be the major suppliers in terms of HIV and AIDS education, care and other services. Churches can aid in the fulfilment of both material needs and spiritual matters and that means they can also be of significant help in combating HIV and AIDS (Errikson et al. 2014). They can provide the community with spiritual counselling, prayers, social and material support. According to the study conducted in Cameroon, some churches necessitate HIV testing for couples before they can commence marriage (Akoku et al. 2018). This promotes abstinence and faithfulness to the couple who are intending to get married and support those who are living with HIV and/or AIDS. However, pastors assume a vital role in providing soothing counselling that there is life after being infected with HIV. KwaZulu-Natal (KZN) has the highest prevalence of HIV and AIDS, which increased from 15.7% in 2002 to 27.9% in 2012 (Padayatchi et al. 2010). Therefore, the study aimed at looking into the roles played by churches in HIV prevention among youths at Nqutu in KZN.

## Study research methodology

### Research design

Qualitative descriptive phenomenology design was used to conduct the study. According to Harvey and Land (2017) a qualitative research methodology allows the researcher to obtain a clear understanding of behaviours, attitudes, interactions, beliefs, experiences and opinions of individuals or groups of people.

### Setting

The study was conducted at Nqutu, which is a small town situated in uMzinyathi District Municipality in the North of KwaZulu-Natal Province in South Africa. uMzinyathi District Municipality is situated approximately 53 km east of Dundee. The study setting was chosen because no study was conducted in this area regarding the phenomenon. The area is 1962 km^2^ and in 2016 it had a total estimated population of 171 325 people (Polit & Beck 2012). The town is made up of rural areas and/or villages and the racial makeup consists of 97.9% black Africans, 0.3% mixed race people, 0.2% Indian people and 0.2% white people (Polit & Beck 2012).

### Population and sampling strategy

For this study, the targeted population consisted of the youth churchgoers between the ages of 18 and 35 years who reside in the selected community at Nqutu. All youth who are churchgoers were included in the sample depending on data saturation, and all youth who are non-churchgoers were excluded. Purposive sampling was used to select the sample for the study. A purposive sampling refers to a non-probability sample, which is chosen based on the features of the given population and based on the main objectives that the study seeks to explore (Greene 2015). The size of the sample was determined by data saturation. Data saturation is the point at which new data appears to no longer contribute to the findings because of repetition of themes and comments by participants. The researcher targeted about 20 participants in this study.

### Data collection

Unstructured descriptive phenomenological interviews were performed. Descriptive phenomenology was chosen for this study because the study was aimed at ‘uncovering and describing the essence of the phenomena of interest’. The grand tour question asked was: ‘Tell me more about your views and experiences regarding the roles of churches in HIV prevention among youth at Nqutu in KwaZulu-Natal?’. This was followed by probing questions to get rich information from the participants. Data were collected between July 2021 and September 2021. Data were gathered by interviewing research participants on the face-to-face encounters in their respective homes. The interviews lasted between 30 min and 45 min. Interviews were conducted in both English and isiZulu as per the participants’ preferences and those who preferred to be interviewed in isiZulu had their transcripts transcribed to English during data analysis. The researched conducted 13 interviews, followed by additional two interviews to determine data saturation, which made the total of 15 interviews. The interviews were audiotaped, and field notes were also taken to ensure rich information is captured. Data collection took place until a point of saturation, which was reached after 15 interviews. The researcher adhered to the coronavirus disease 2019 (COVID-19) regulations during data collection by ensuring that 1.5 m distance between the sitting was adhered to, masks were worn by the researcher and the participants including the use of hand sanitisers.

### Data analysis

Data were manually analysed concurrently with data collection. The seven steps of Colaizzi’s method of data analysis for descriptive phenomenological research were applied, which included familiarisation of data by reading through and understanding all the participant accounts several times; identifying significant statements of direct relevance to the phenomenon under investigation; formulating meanings by recognising related phenomenon that arise from carefully consideration of the significant statements; clustering themes grouping together the identified meanings into themes that are mutual across all accounts; developing an exhaustive description writing a full and comprehensive description of the phenomenon; producing the fundamental structure by summarising the exhaustive description down to a short and dense statements capturing aspects deemed to be essential to the structure of the phenomenon; and seeking verification of the fundamental structure by returning the fundamental structure statement to a subsample of participants to ask whether it captures their experiences (Lincoln & Guba 1985).

### Measures to ensure trustworthiness

The proposed four criteria for developing the trustworthiness of a qualitative study, namely credibility, dependability, confirmability and transferability, were adhered to in this study (Lincoln & Guba 1985). These criteria were adhered to in this study to authenticate the researcher’s loyalty and honesty to the participants during the research study. To ensure credibility, all interviews were audiotaped, and field notes were taken. Bracketing was performed to ensure that presumptions of the researcher were excluded. All transcribed data were sent to both study promoters for further analysis to ensure confirmability. Dependability was ensured by safeguarding that all processes in the study were reported in detail so that an external researchers could repeat the study to obtain similar findings. Transferability was established by describing the research methods.

## Findings of the study

Table 1 represent the demographic data of the participants. Several themes and sub-themes emerged from the study interviews and are summarised in Table 2.

**TABLE 1:** Demographic data of participants.

Participants	Age (years)	Gender	Race	Marital status	Religion	Level of education	Employment status
AP1	30	Female	African	Single	Christian	Higher certificate	Employed
AP2	20	Male	African	Single	Christian	Diploma	Employed
BP2	32	Female	African	Single	Christian	Higher certificate	Employed
BP3	23	Male	African	Single	Christian	Diploma	Employed
CP3	21	Female	African	Single	Christian	Grade 12	Unemployed
CP4	28	Female	African	Single	Christian	Degree	Unemployed
DP4	31	Female	African	Single	Christian	Diploma	Employed
DP5	36	Female	African	Single	Christian	Degree	Employed
EP5	22	Female	African	Single	Christian	Grade 12	Unemployed
EP6	33	Male	African	Single	Christian	Grade 12	Unemployed
FP6	27	Male	African	Single	Christian	Degree	Employed
GP7	21	Male	African	Single	Christian	Degree	Employed
GP8	19	Female	African	Single	Christian	Grade 12	Unemployed
EP9	19	Male	African	Single	Christian	Grade 12	Unemployed
CP10	23	Female	African	Single	Christian	Degree	Employed

**TABLE 2:** Themes and sub-themes.

Themes	Sub-themes
1. Churches’ contribution to HIV prevention	1.1. Contribution of youth groups in HIV prevention 1.2. HIV testing, counselling and care
2. Health awareness	2.1. Commemoration of World AIDS Day
3. Churches’ involvement in sexual education	3.1. Abstinence 3.2. Faithfulness 3.3. Condom use 3.4. Prevention, support and treatment
4. Churches’ partnership with stakeholders	4.1. Partnership with Department of Health 4.2. Church and community leaders’ partnership
5. Financial resources	5.1. A lack of financial resources

HIV/AIDS, human immunodeficiency virus and acquired immunodeficiency syndrome.

### Theme 1: Churches contribution to HIV prevention

The church is perceived as an important institution and a place of safety. Most participants stated that a church exerts a powerful influence in the communities where they operate and has credibility in society, and in that way, it can influence the decision-making of the youth in preventing the spread of HIV and/or AIDS.

#### Sub-theme 1.1: Contribution of youth groups in HIV prevention

Most of the participants verbalised that in church they have youth groups that are led by their fellow youth leaders. These youth groups help to keep them occupied and away from the streets, where they might find themselves involved in wrongdoings. During youth activities at church, the groups engage in different topics, including HIV and/or AIDS. The youth leaders guide debates, discussions and understanding of what HIV or AIDS is, including how they can prevent themselves from being infected. This is evident in the following statements:

‘In my church we have youth meetings every Saturdays after a normal church service. In those meetings we are led by youth leaders, and we discuss about issues that affects us as youth like HIV and AIDS … We are taught about the benefits of HIV prevention like having strong immune system and better quality of life to eliminate chances of being sick.’ (P1, Female, 22 years)

Other participant with same opinion stated that:

‘Uh …Yes, we do hold youth groups’ meetings every Wednesday from 18H00 to 19H30 in the evening where we have a session called Life skills … As youth we meet and talk about different topics including HIV and we learn about the importance of prevention against HIV.’ (P4, Male, 24 years)

#### Sub-theme 1.2: HIV testing, counselling and care

The research participants shared that some of their churches value the importance of HIV testing, counselling and care. They believed it encourages youth in the church to know about their HIV status. Other participants verbalised that their churches are used as testing sites in their communities. This was extracted from the following excerpts:

‘Our church is often used as a testing site for HIV by health care care workers from Department of Health. They usually come and place their tents outside our church and provide services like HIV testing and counselling that means our church play a role in HIV prevention.’ (P12, Female, 26 years)

Another participant had to say:

‘… Mam … we do receive HIV testing and counselling including support at our church. The health care workers during their visit at our church they encourage us to do tests for HIV so that we know our statuses.’ (P11, Male, 20 years)

### Theme 2: Health awareness

During the study interviews, the participants mentioned that in their churches they do have health awareness where information about HIV and AIDS is disseminated to the church members, mostly the youth.

#### Sub-theme 2.1: Commemoration of world AIDS Day

Participants revealed that World AIDS Day gives them the opportunity to unite as a church in the fight against HIV and/or AIDS and to raise awareness about the existence of HIV and AIDS. Also, show support for people living with HIV and to commemorate those who died because of AIDS-related illness. Concerning the World AIDS Day, most participants verbalised that in their churches they invite healthcare workers or people who are knowledgeable about HIV and/or AIDS to come and teach the youth church members. This was apparent in the following account:

‘Um … During the World AIDS Day in my church, the leaders invite the HIV/AIDS activists who have lived with the disease for many years to educate us about the importance of HIV/AIDS prevention and how to take care of yourself if you are already infected.’ (P13, Female, 29 years)

Another participant said that:

‘In my church we commemorate the World AIDS Day by lighting candles in memory of those that died due to AIDS-related illness, we also engage in discussions about how to prevent the spread of HIV. Sometimes there are speakers invited to teach us about HIV prevention and support.’ (P2, Male, 30 years)

### Theme 3: Churches’ involvement in sexual education

Most participants verbalised that sexual education is a common subject in their churches, especially during youth sessions.

#### Sub-theme 3.1: Abstinence

Abstinence was explained by the participants as the practice of refraining from sexual activity. Most of the participants concurred that the church has historically encouraged and guided youth in practicing abstinence prior to marriage. The participants verbalised that their pastors and elders preach and encourage abstinence from sexual activities during their youth groups’ discussions, which assists them to learn abstinence as an effective method of HIV prevention. This was extracted from the following excerpt:

‘… Uh … Another good thing about our church is that our Pastor teaches youth about the benefits of abstaining from sexual practices to reduce the risk of contracting HIV.’ (P7, Female, 26 years)

The other participant said:

‘In my church the elders are very strict when it comes to sexual education, they always teach us about abstaining from sexual activities until we are married. They say that abstinence can prevent us from unplanned pregnancies and HIV and AIDS.’ (P1, Female, 21 years)

#### Sub-theme 3.2: Faithfulness

The research participants verbalised that in their churches they are taught about being faithful in relationships. Most of the participants verbalised that the couples who are about to get married are also taught during counselling sessions about being faithful to one another in their marriages to prevent contracting diseases such as AIDS. This was apparent in the following account:

‘In my church we are taught about the importance of faithfulness in life generally and faithfulness to one’s partner. Married couples are taught that extramarital relationships can lead to contracting HIV, therefore, being faithful to one partner is important.’ (P9, Female, 23 years)

Another participant alluded that:

‘… Uh … Our church condemns promiscuity, the church encourages those who are promiscuous to repent, therefore, as youth we are taught about faithfulness. These teachings are important to curb the spread of HIV, especially if both partners can be faithful to one another.’ (P15, Male, 31 years)

#### Sub-theme 3.3: Condom use

The participants verbalised that they are taught about condoms and condom usage in their churches by the healthcare workers who conduct youth programmes and some pastors and church leaders do teach youth about condom use. This was evident in the following statements:

‘… Uh … During the HIV and AIDS awareness campaigns, in my church the church leaders and health care workers do teach us about using a condom when engaging in sexual activities for those who cannot abstain, but the church strongly discourages engaging in sexual intercourse before marriage to prevent being infected by HIV and other diseases.’ (P14, Female, 25 years)

Another participant alluded:

‘In my church … Mam, we are encouraged by church leaders that if you are sexually active, you should make sure that you use a condom during sexual activities because when condoms are used correctly and consistently they are a reliable method of protecting both partners from HIV and other sexually transmitted illnesses.’ (P10, Male, 30 years)

#### Sub-theme 3.4: Prevention, support and treatment

It was evident from the responses of the participants that church leaders are in the unique position of being able to preach the positive messages on how to prevent HIV and/or AIDS because they can shape social values and increase church members’ knowledge not only about HIV prevention but also support and taking treatment for those that are already infected by HIV-related disease. This was extracted from the following excerpt:

‘You know … Uh … I can say that a church is important in disseminating educational information on HIV prevention. During the youth sessions we learn about the importance of accepting, welcoming and supporting those who have disclosed that they are already infected by HIV without discriminating them, these talks help in reducing the new HIV infections among youth.’ (P2, Male, 30 years)

Another participant with the same view said:

‘Our Pastor and other elders of the church always advocates prevention and support among those who are living with HIV or AIDS, youth is also encouraged that if one is HIV positive, they should not be ashamed of taking treatment.’ (P9, Female, 23 years)

### Theme 4: Churches’ partnership with stakeholders

The most practical approach to combating the effects of HIV and/or AIDS is to encourage collaboration and networking with other stakeholders. Partnerships between marginalised communities and support organisations such as churches, the public sector, the private sector and civil society are critical components of HIV and/or AIDS management policy.

#### Sub-theme 4.1: Partnership with the Department of Health

Most study participants stated that they invite health workers to lead discussions in HIV and AIDS awareness youth workshops in their churches. This is important because healthcare workers are highly trained in the field of HIV and/or AIDS, so they can give accurate information to the youth in churches. This is extracted from the following statements:

‘Yes, other health care workers are members of our church but if we have big conferences or awareness campaigns, our Pastor invite managers of the nearest clinic and they send health care workers to come and share HIV and AIDS related information.’ (P6, Female, 24 years)

Another participant also added:

‘In my church, our leaders requested the health care workers to give us pamphlets that contained HIV prevention methods, this initiated a good relationship with our church and the health care workers from our nearest clinic in such a way that they also helped us to start an HIV and AIDS awareness campaign in our church.’ (P3, Male, 20 years)

#### Sub-theme 4.2: Church and community leaders’ partnership

The participants verbalised that churches exert a powerful influence in the communities where they operate and have credibility in society. Therefore, the partnership of community leaders and church leaders is crucial in communicating the HIV prevention message to the youth. Some participants mentioned that their Pastors engage with community leaders to be able to reach out to the youth in their communities and spread the HIV prevention messages. The participants shared their experiences on this in the following statements:

‘Uh … We all know that the church is situated within the community, so the church leaders liaise with the community leaders every time when there will be HIV and AIDS awareness campaigns in church. The community leaders share that information with the youth in the community during the community meetings. This partnership helps to spread HIV prevention information to more youth in the community.’ (P8, Female, 33 years)

Another participant also added:

‘Both the church and the community leaders’ partnership help in reaching the large masses of youth in the community. In my church, the Pastor communicates with the community leaders when the Department of Health is planning to come to provide HIV related services, and the community leader invites other youth in the community who are not our church members to attend the sessions.’ (P14, Female, 25 years)

### Theme 5: Human and financial resources

Most of the study participants concurred that the church, just like any other organisations need human and financial resources to function properly.

#### Sub-theme 5.1: A lack of financial resources

Most churches rely on the generous donations of its members. Other participants also mentioned that they have limited financial resources, which makes it difficult to maintain their HIV and/or AIDS prevention programme. This was apparent in the following statements:

‘The only source of funding that we have in my church is the donations from the church members but most of them are unemployed, so sometimes we lack money to conduct and maintain HIV prevention programs.’ (P3, Male, 20 years)

‘What I have noticed in my church is that the funds that are given to the youth to support their programs are always insufficient. I think the problem is due to the unavailability of funds. Therefore, we end up cancelling other events because of the lack in financial backing.’ (P13, Female, 29 years)

## Discussion

### Churches’ contribution to HIV prevention

The study participants mentioned that during youth group meetings, their youth leaders help them to debate, discuss and understand what HIV and AIDS is, including how they can prevent themselves from being infected. Approximately, 73% of the young people who were interviewed in another study mentioned that they had received information about sexuality and HIV and/or AIDS in church youth groups and that their leaders were reliable as teachers in sex education (Errikson et al. 2014). Church youth groups are crucial for gaining relevant and up-to-date information about HIV and AIDS and, in turn, lead to behavioural change (Ochillo et al. 2017). When young people partake meaningfully in the development and implementation of programmes that affect their health, services are more efficiently tailored to their needs and their health outcomes improve (UNAIDS 2018). Likewise, development programmes that focus on youth are most effective when it is the youth who contribute to executive spaces about interventions that affect their lives and their contributions are meaningfully considered (UNAIDS 2018). Church youth groups are important for disseminating HIV prevention information. Therefore, church-going youth should be encouraged to attend youth groups and to participate in HIV and AIDS education.

Most of the participants believed the church contributes to HIV prevention by advocating for HIV testing, counselling and care, which lead to more youth become aware of the prevention measures against HIV. Their opinions were that it encourages youth in the church to know about their HIV status. Other participants verbalised that their churches are used as testing sites in the communities. Churches that conduct HIV prevention programmes provide pre- and post-counselling, which provides emotional support and care to the youth (Van Dyk 2017). It was found that encouraging people to get tested is part of the good ordinary acts of churches counselling in Khayelitsha, Cape Town, South Africa. Many churches also collaborate with local clinics by sending counsellors to their testing sites (Burchardt 2013). The prevailing purposes of the pre-HIV testing sessions in the church are to prepare youth for the HIV test and to support their conviction that they are doing the right thing despite what the results might be (Derose et al. 2016). This shows that most churches are involved in HIV counselling, testing and care.

### Health awareness

World AIDS Day is a chance for the church to fight against the dreadful disease, preconception and discrimination that affect those living with HIV and curb the spread of the disease. Many churches collaborated to jointly host events to mark the National HIV and AIDS Testing Day and World AIDS Day (Coleman et al. 2016). Church leaders use the opportunity provided by special awareness days such as World AIDS Day to talk to the youth about sexuality and HIV prevention, acceptance and care of those leaving with the disease (Van Dyk 2017). Therefore, it is evident that churches are involved in teaching youth about the prevention of the spread of HIV by commemorating World AIDS Day and teaching the youth about HIV and AIDS.

### Churches’ involvement in sexual education

The main HIV prevention message that most pastors are willing to share is abstinence or ‘your body is the temple of God’ only (Van Dyk 2017). Scholars who work in the fields of religion, youth and sexuality agree that the main message of the church to young people on how to curb the spread of HIV infection is commonly a message of ‘abstinence only’ before marriage (Van Dyk 2017). In the study that was conducted in Uganda, the Pentecostals voiced that abstinence messages can lead to reduced HIV infections (Bompani & Brown 2015). Moreover, according to the Catholic Church doctrine, sex is only permitted within the context of marriage (Vatican 1976). It emanated to this study participants that church leaders do advocate for abstinence to promote the prevention of HIV among youths. Likewise, it was found in another study that most participants (approximately 85%) reported that their church leaders advocate for abstinence (Munguri et al. 2021). Therefore, abstinence is perceived by the church as an effective method of HIV prevention among youth.

The study participants verbalised that in their churches they were taught about being faithful in relationships. This was attested by most of the participants that in their churches, couples who are about to get married are taught during counselling sessions about being faithful to one another in their marriages to prevent contracting HIV. Most church leaders encourage their members to be faithful in their relationships to prevent being infected by HIV (Van Dyk 2017). Other scholars further agree with the notion that the church leaders’ advocate for faithfulness as one of the HIV prevention strategies, as being faithful among the married was the most encouraged HIV prevention strategy by religious leaders (Munguri et al. 2021). Generally, the Pentecostal churches in Botswana have highlighted sexual fidelity within marriage or faithfulness as a strategy to avoid HIV infection (Mpofu et al. 2014). Therefore, it is evident that sexual education is discussed in churches where faithfulness is one of the most encouraged methods of preventing HIV.

Although there is a controversy about the issue of condom use in churches as an HIV prevention method, the participants of this study verbalised that they are taught about condoms and their use in their respective churches by the healthcare workers who conduct youth programmes and some pastors. Church leaders are willing to disseminate sex education, including the use of condoms, to their youth. Approximately, 80% of church leaders reported that condoms prevent HIV, and they passed that message to their congregants (Ralte 2021). This might send negative message to the youth that they should engage in sexual activities using condoms, which might also carry some risk of contracting HIV in case the condom is not correctly used or if it bursts. Some church leaders can advise their congregants, especially, youth about the use of condoms even though they said, ‘it is against the beliefs of their religion’ (Munguri et al. 2021).

Furthermore, condom use is the most reported sexual behaviour, and some church leaders have approved condom use for their church members with caution (Ochillo et al. 2017). Despite the views that condom use is against the belief of most churches, church leaders are attempting to teach youth about condoms in churches to reduce the spread of HIV.

Prevention, support and treatment were also mentioned by the research participants as the strategy of the church for combating the spread of HIV. Most religious leaders expressed their willingness to contribute to HIV prevention and give support to those who are already infected by the disease (Ralte 2021). Church leaders have implemented a variety of activities, including encouraging people to seek treatment, providing care and support to people living with HIV and disseminating prevention messages to the youth (Williams et al. 2018). The church does not only preach about HIV prevention but also cares for those who are already infected and encourages them to take their treatment.

### Churches’ partnership with stakeholders

The study participants indicated that there is a partnership between their churches and the department of health.

They invite the health workers to lead the discussions in HIV and AIDS awareness youth workshops in their churches.

In Germany, the African pastors collaborated with the AIDS service organisations, public health authorities and researchers in a community-based participatory health project that aims at combating the spread of HIV among youth (Gangarova & Bakambamba 2019). It was also evident from the participant’s responses that in some churches there are HIV and AIDS programmes that were initiated with the help of the healthcare workers from the Department of Health. Similarly, a mobile training sequence on HIV and AIDS for African pastors was established with the support of medical doctors (Gangarova & Bakambamba 2019). There is a remarkable public health opportunity to work collaboratively with churches to address the HIV epidemic in their communities (Nuun, Jeffries & Foster 2019). Pastors are taking a much stronger role in making lives of youth bearable, giving them hope and providing them spiritual support (Williams & Brewer 2019). Therefore, it is important that the churches seek information regarding HIV and/or AIDS from the Department of Health.

Most study participants alluded that their churches are influential in engaging youth in HIV and AIDS programmes in partnership with community leaders such as ward councilors. Some participants mentioned that their pastors engage with community leaders to be able to reach out to the youth in their communities and spread the HIV prevention messages. Various collaborative, community-based associations involving community leaders and churches have been successful in promoting positive health behaviours and preventing the spread of HIV among youth (Williams & Brewer 2019). In both the United States and in African countries, ordained priests and community leaders are beginning to work together to form faith-based programmes to reduce HIV and reduce the stigma that is associated with the disease (Nehl et al. 2016). Church leadership and community partnership are imperative in the reduction of HIV among the youth within the church and the community at large. Therefore, the Department of Health should consider partnering with religious leaders and community leaders to encourage more youth to undergo HIV testing and, in turn, reduce the spread of HIV.

### Financial resources

Every organisation, including churches, needs human and financial resources to function properly. Most churches are going through financial difficulties as they do not have a stable financial resource but mostly depend on donations from good Samaritans and some members of the church. The study participants mentioned that they have limited financial resources, which makes it difficult to maintain their HIV prevention programmes. Acquiring financial resources makes it feasible for pastors to implement HIV prevention activities (Pichon et al. 2016). A lack of financial support was mentioned by the church leaders as one of the barriers that hinders the implementation and maintenance of HIV prevention programmes (Robinson et al. 2018). Therefore, it is evident that to sustain and implement a successful HIV programme for the youth at churches requires sufficient financial support.

## Strength and limitations

The findings of this study are relevant, as this is the first to be carried out on the youth of Nqutu in KZN. The importance of the involvement of church organisations in the prevention of HIV among youth cannot be overemphasised. A qualitative descriptive and explorative phenomenological study was conducted using unstructured interviews with the participants who were purposefully selected. Therefore, the findings of this study cannot be generalised because the sampling size was relatively small. Furthermore, only the youth at selected churches in Nqutu were included in the study, the study could have yielded different findings if the non-churchgoers’ youth, pastors, church leaders and the congregation formed part of the inclusion criteria for the study sample.

## Recommendations

The church leaders and/or pastors need to strengthen their collaboration with stakeholders such as the Department of Health, Non-Government Organisations and the community to ensure that their programme regarding HIV prevention among the youth are sustainable. Church leaders should seek out relevant up to date HIV and AIDS information to be given to the youth and the congregations from the Department of Health to ensure that the information is current and relevant. It is recommended that community leaders should work together with the church leaders and/or pastors in the fight against HIV and AIDS to promote and sustain available youth programme in the community and in church. Furthermore, youth should attend and actively participate in HIV prevention activities. The Department of Health should organise workshops to keep religious leaders abreast of the new developments in HIV prevention. Moreover, youth friendly policies should be developed and implemented regarding the prevention of HIV. Further research is required to document the outcomes of the work performed by churches in HIV prevention.

## Conclusion

The purpose of the study was to explore the roles of churches in HIV prevention among youth at Nqutu in KZN and to describe the factors influencing the involvement of churches HIV prevention. The study’s findings revealed that churches do play a role in curbing the spread of HIV, especially among church-going youth. The programmes such as youth groups, awareness days and teachings by the Pastors and elders available in most churches play important roles among the youth in curbing the HIV epidemic. Furthermore, the findings of the study achieved the aim and objectives of the study and revealed that churches play an important role in engaging the youth regarding HIV prevention and support strategies.
